# TCF-1 participates in the occurrence of dedifferentiated chondrosarcoma

**DOI:** 10.1007/s13277-016-5235-3

**Published:** 2016-08-13

**Authors:** Xiaolong Xu, Xiaodong Tang, Wei Guo, Kang Yang, Tingting Ren

**Affiliations:** 1 Musculoskeletal Tumor Department and Beijing Key Laboratory of Musculoskeletal Tumor, Peking University People’s Hospital, Beijing, China; 2 Musculoskeletal Tumor Center, Peking University People’s Hospital, No. 11 Xizhimen South Street, Beijing, 100044 China

**Keywords:** Chondrosarcoma, Dedifferentiated chondrosarcoma, T cell factor-1, Runt-related transcription factor 2, Sry-related HMG box 9, Prognosis

## Abstract

The present study demonstrated that T cell factor 1 (TCF-1) protein, a component of the canonical Wnt/β-catenin signaling pathway, can regulate the expression of runt-related transcription factor 2 (*runx2*) gene and Sry-related HMG box 9 (*sox9*) gene, which may participate in the differentiation of chondrosarcoma. Dedifferentiated chondrosarcoma (DDCS) is a special variant of conventional chondrosarcoma (CCS), associated with poor survival and high metastasis rate. However, little is known about the mechanism of its occurrence; thus, no effective treatment is available except surgery. Earlier, high expression of *runx2* and low expression of *sox9* were found in DDCS compared with CCS. Using Western blot to detect clinical tissue samples (including 8 CCS samples and 8 DDCS samples) and immunohistochemistry to detect 85 different-grade chondrosarcoma specimens, a high expression of TCF-1 in DDCS tissues was found compared with CCS tissues. This difference in expression was related to patients’ prognosis. Results of luciferase, chromatin immunoprecipitation, and gel electrophoresis mobility shift assays demonstrated that TCF-1 protein could bind to the promoter of *runx2* gene directly and *sox9* gene indirectly. Hence, it could regulate expression of *runx2* gene positively and *sox9* gene negatively. Furthermore, in vitro and in vivo experiments showed that TCF-1 protein was closely related to the phenotype and aggressiveness of chondrosarcoma. In conclusion, this study proved that TCF-1 participates in the dedifferentiation of DDCS, which may be mediated by *runx2* gene and *sox9* gene. Also, TCF-1 can be of important prognostic value and a promising therapeutic target for DDCS patients.

## Introduction

Chondrosarcoma is a common primary bone malignancy. Conventional chondrosarcoma (CCS) consists of three histological grades [1–3]. Also, a special kind of chondrosarcoma exists called dedifferentiated chondrosarcoma [4]. It was first reported by Dahlin and Beabout in 1971. It is characterized by its special components: a low-grade chondrosarcoma (CCS) and a high-grade malignant tumor (i.e., osteosarcoma, undifferentiated pleomorphic sarcoma, etc.), with an abrupt interface between them [5, 6]. This kind of tumor is associated with a high recurrence rate, a high metastasis rate, as well as poor prognosis. Most patients die of multiple metastases within 2 years after disease onset [7]. Till now, little is known about the origin of dedifferentiated chondrosarcoma and its mechanism. Bovee et al. reported that the two different components of dedifferentiated chondrosarcoma (DDCS) may derive from the same precursor, but the cause of the difference in differentiation remains unknown [8, 9]. Since no effective therapy is available except surgery [4, 7], it is urgent to explore a novel therapy for dedifferentiated chondrosarcoma.

Canonical Wnt signaling pathway is a highly revolutionarily conserved pathway that participates in a variety of biological processes [10]. This pathway plays a key role in the differentiation of messenchymal cells into osteocytes and chondrocytes via runt-related transcription factor 2 (*runx2*) gene [11, 12]. Canonical Wnt signals are transduced through frizzled (FZD) family receptors and low-density lipoprotein receptor-related protein (LRP)5/LRP6 co-receptor to the β-catenin signaling cascade. In the presence of canonical Wnt signaling, disheveled (DVL) is phosphorylated and β-catenin is then released from phosphorylation for stabilization and nuclear accumulation. Nuclear β-catenin forms a complex with T cell factor/lymphoid enhancer factor (TCF/LEF) family transcription factors and activates the transcription of target genes [13]. TCF/LEF is the key factor of canonical Wnt signaling, playing vital roles in the formation of many kinds of tumors (i.e., gastrointestinal tumors, lymphoid hematopoietic tumor, etc.) [14–18]. TCF/LEF family consists of four members: TCF-1, LEF-1, TCF-3, and TCF-4 [13]. These factors participate in a variety of diseases, especially in tumor formation [14–18]. The T cell factor 1 (TCF-1) is a molecular switch for the differentiation of bone or cartilage by interacting with *runx2* gene [11, 12]. In some previous studies, the researchers found that *runx2* and Sry-related HMG box 9 (*sox9*) may participate in the dedifferentiation of chondrosarcoma, but the specific mechanisms are still unknown [19–21]. A previous study demonstrated the difference in the *sox9* and *runx2* expression between DDCS and CCS cell lines [22]. But what causes the change in *sox9* and *runx2* expression remains unknown.

In the present study, clinical samples were tested, in vitro and in vivo tests were performed, and it was found that TCF-1 was related to the patients’ prognosis and the invasiveness of DDCS. Then, the specific mechanism of the participation of TCF-1 in the occurrence of DDCS was investigated. This study found a more specific mechanism of DDCS formation that had never been reported earlier. The results showed that TCF-1 could be a marker of prognostic value. More importantly, it could be a promising therapeutic target for DDCS, for which no effective therapy is available yet except surgery.

## Results

### TCF-1 expression is correlated with the clinicopathological features of chondrosarcoma, especially dedifferentiated chondrosarcoma. Also, it predicts the treatment outcome

**Fig. 1:**
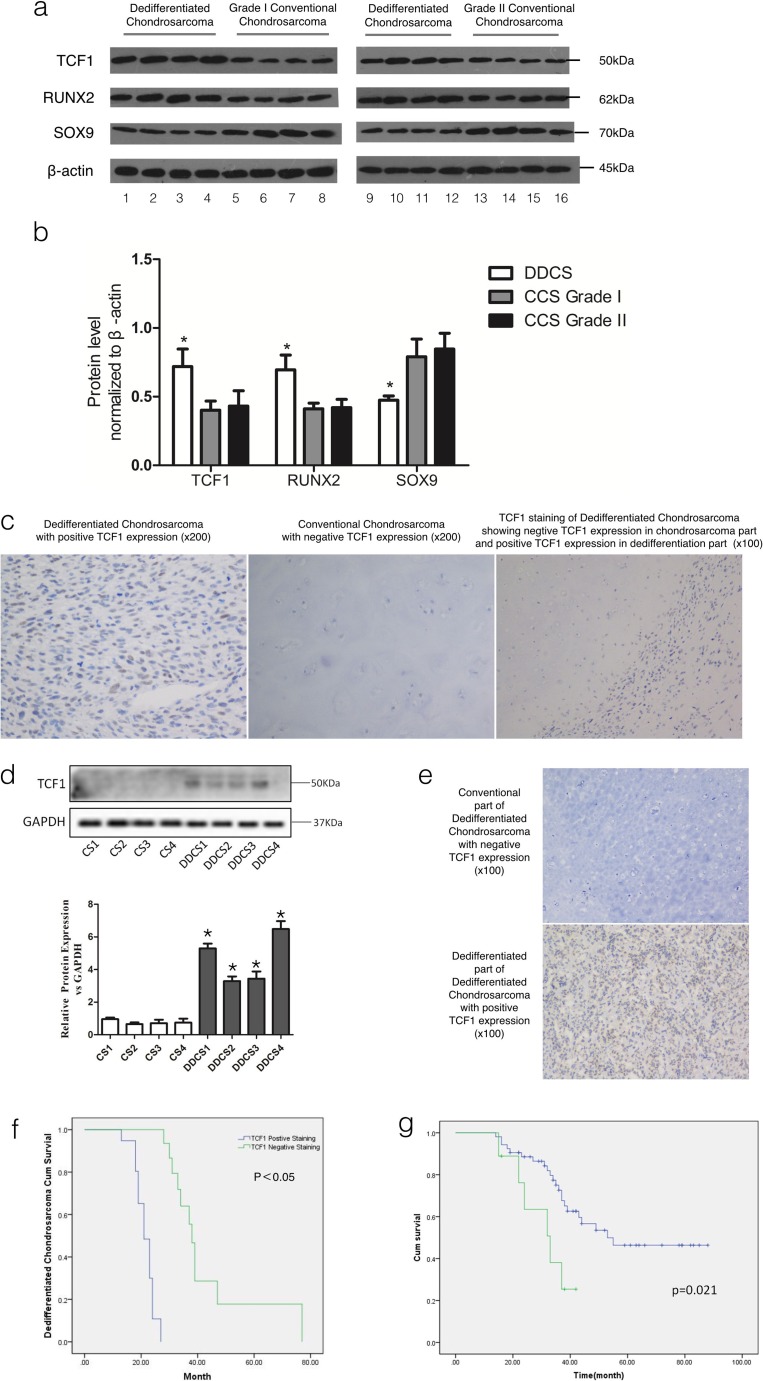
TCF-1 expression is correlated with clinicopathological features of chondrosarcoma, and predicts treatment outcome. The Western blot analysis showed that TCF-1 was highly expressed in dedifferentiated chondrosarcoma compared with normal chondrosarcoma. *Lanes 1*–*16* are as follows: *lanes 1*–*4*, *9*–*12* dedifferentiated chondrosarcoma; *lanes 5*–*8* grade I conventional chondrosarcoma; *lanes 13*–*16* grade II conventional chondrosarcoma. **a**, **b** The expression of TCF-1 in a cohort of 85 human chondrosarcoma specimens was determined by IHC staining. **c** Representative images of TCF-1 IHC staining in conventional and dedifferentiated chondrosarcoma specimens are shown. **d** The expression level of TCF-1 in the conventional chondrosarcoma part as well as dedifferentiated part is tested using Western blot. CS1 and DDCS1 are the two parts of the same dedifferentiated chondrosarcoma sample, so are CS2 and DDCS2, CS3 and DDCS3, and CS4 and DDCS4. **e** IHC also shows that in the different part of the same dedifferentiated chondrosarcoma specimen, TCF-1 expression level differs. **f** The expression level of TCF-1 was in correlation with the patients’ prognosis. Kaplan–Meier curves in chondrosarcoma patients with or without positive TCF-1 staining. Positive TCF-1 staining (>10 % cells stained with TCF-1) in chondrosarcoma specimens is significantly associated with a poorer prognosis (*P* < 0.050). **g** Follow-up of conventional chondrosarcoma patients showed that positive expression of TCF-1 indicated poorer relapse-free survival (*P* = 0.021). Data are representatives or expressed as the mean + standard error of mean of three independent experiments. *Asterisk* denotes *P* < 0.05

The expression level of TCF-1 is higher in DDCS than in CCS. Western blot was performed with eight DDCS specimens (dedifferentiated part confirmed by pathology) and eight CCS specimens to investigate the expression level of TCF-1, RUNX2, and SOX9. The results showed that the expression level of TCF-1 and RUNX2 was higher in DDCS than in CCS, while the expression level of SOX9 was lower in DDCS than in CCS (Fig. 1a, b). Then, immunohistochemistry (IHC) staining was performed in 85 different-grade chondrosarcoma specimens to assess the expression level of TCF-1. A total of 25 % of tumor samples were positive [23] for TCF-1 staining. The results showed that TCF-1 expression level was higher in DDCS than in CCS, especially in the dedifferentiated portion of DDCS. Representative TCF-1-positive and TCF-1-negative staining images were shown in Fig. 1c. Also, we tested four DDCS specimens, compared the expression level of TCF-1 between their dedifferentiated part and their conventional chondrosarcoma part. Using Western blot and IHC, we found that TCF-1 expression level is much higher in dedifferentiated part than conventional part in DDCS specimens (Fig. 1d, e). The correlation between TCF-1 expression and the clinicopathological parameters of chondrosarcoma patients was analyzed. As summarized in Table 1, TCF-1 expression was detected as low-grade chondrosarcoma in 2 of 29 patients (grade I), high-grade chondrosarcoma in 7 of 33 patients (grade II + III), and dedifferentiated chondrosarcoma in 12 of 23 patients. The TCF-1 expression level was significantly higher in DDCS specimens than in CCS specimens (*P* = 0.001). In addition, using Kaplan–Meier survival analysis (*P* < 0.05), it was found that expression level of TCF-1 was related to patients’ survival rate. The prognosis analysis showed that the rate of survival in dedifferentiated chondrosarcoma patients was poor in cases with higher TCF-1 expression level, indicating that TCF-1 could be a prognostic marker for dedifferentiated chondrosarcoma (Fig. 1f). Besides, positive staining of TCF-1 in conventional chondrosarcoma indicated poorer relapse-free survival (Fig. 1g).

**Table 1:** The relationship between TCF-1 expression and clinicopathological variables of chondrosarcoma

Clinopathological variables	No. of cases	TCF-1 expression		*P* value
		Positive	Negative	
Sex				0.953
Male	45	11	34	
Female	40	10	30	
Age				0.961
≥40 years	53	13	40	
<40 years	32	8	24	
Anatomical location				0.758
Limb bone	34	9	25	
Axial bone	51	12	39	
Grade of tumor				0.001
Low (grade I)	29	2	27	
High (grade II + III)	33	7	26	
Dedifferentiated	23	12	11	

### The knockdown of TCF-1 by Lentivirus-mediated shRNA in DDCS cell line inhibited the growth of dedifferentiated chondrosarcoma cells. The overexpression of TCF-1 by Lentivirus in CCS cell line promoted the growth of CCS cells

**Fig. 2:**
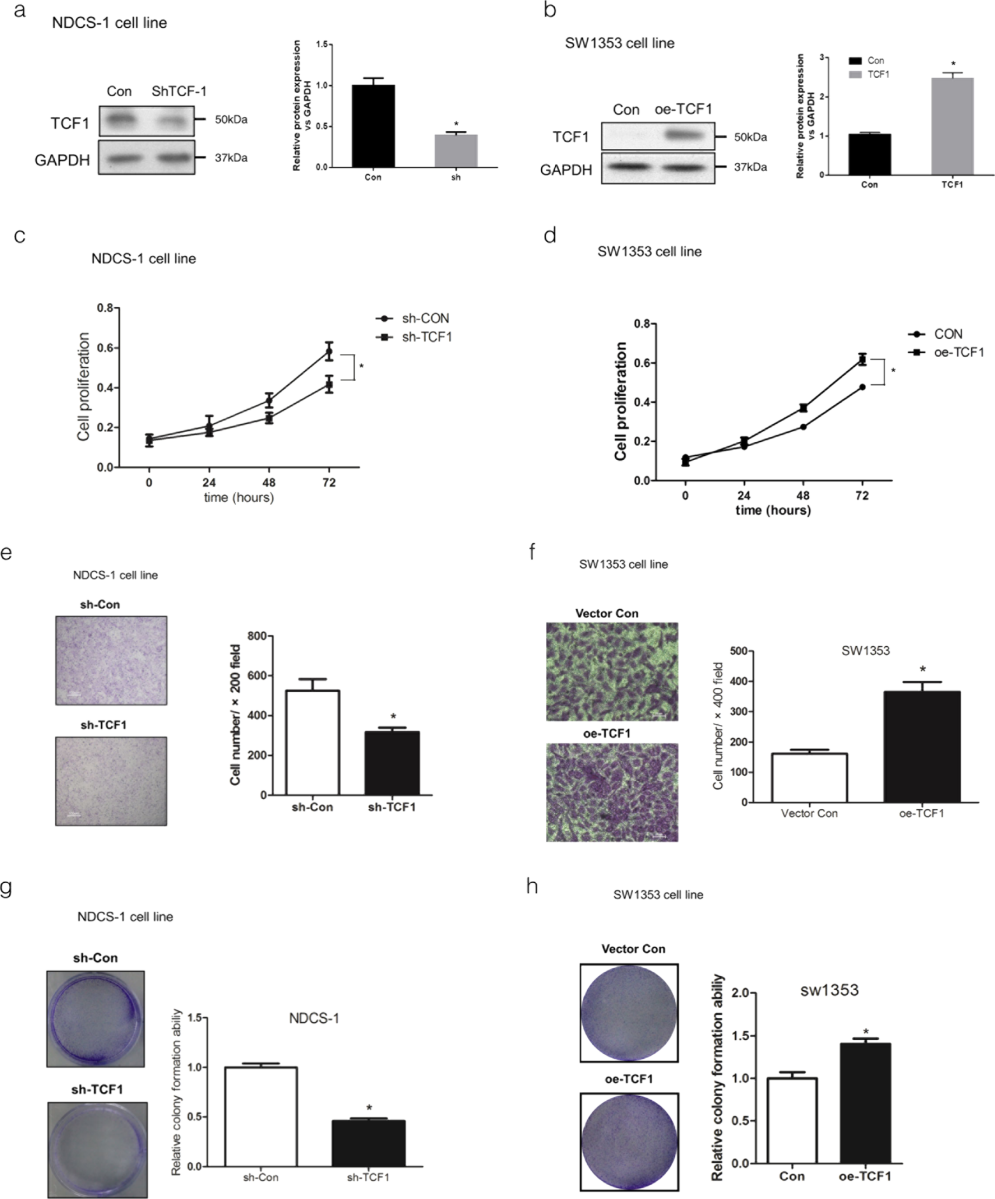
Effects of overexpressed TCF-1 on SW1353 cell lines and downregulated TCF-1 on NDCS-1 cell lines. Expression of protein levels of TCF-1 in NDCS-1 and SW1353 cells were significantly changed at 48 h after shTCF-1 or Lentivirus transfection. The shTCF-1 and Lentivirus transfection efficiency were measured by Western blot (**a**, **b**). The cell viability of NDCS-1 (**c**) and SW1353 (**d**) was determined using the CCK8 assay. The overexpression of TCF-1 could promote SW1353 cell proliferation, while the downregulation of TCF-1 could decrease NDCS-1 cell proliferation. The invasiveness ability of NDCS-1 (**e**) and SW1353 (**f**) cells was examined using the Transwell assay. Images of invasion in NDCS-1 (**e**) and SW1353 (**f**) cells are represented by overexpressed or downregulated TCF-1. The overexpression of TCF-1 could promote the invasive ability of SW1353 cells, while the downregulation of TCF-1 could decrease the invasive ability of NDCS-1 cells. The average number of cells per field was calculated. A significant difference in the number of colonies was expressed between downregulated TCF-1 NDCS-1 (**g**) or overexpressed TCF-1 SW1353 cells (**h**) with the vector control NDCS-1 or SW1353 cells. Data are representatives or expressed as the mean + standard error of mean of three independent experiments. *Asterisk* denotes *P* < 0.05

Lentivirus-mediated small hairpin RNA (shRNA) was used to knock down the expression level of TCF-1, and Lentivirus technology was used to overexpress TCF-1 to investigate the effect of TCF-1 on the maintenance of cell viability in dedifferentiated as well as CCS cell lines. The results revealed that the TCF-1 expression level was suppressed significantly in DDCS cell line (NDCS-1) treated with Lentivirus-mediated shRNA and upregulated in CCS cell line (SW1353) treated with Lentivirus (Fig. 2a, b).

The cell viabilities (detected by Cell Counting Kit-8 (CCK8) assay) decreased sharply after the shTCF-1 transfection in NDCS-1, while they increased after the treatment with Lentivirus in SW1353 cells (Fig. 2c, d). The Transwell assay showed that the overexpression of TCF-1 could promote the invasion ability of SW1353 cells while the inhibition of TCF-1 expression could repress NDCS-1 cell invasion ability (Fig. 2e, f). The colony formation assay showed that owing to the inhibition of TCF-1, the capacities of colony formation of NDCS-1 cells significantly decreased while those of SW1353 cells increased (Fig. 2g, h). Therefore, the repression of TCF-1 inhibited the growth of dedifferentiated chondrosarcoma cells, while the overexpression of TCF-1 increased the growth of CCS cells.

### Results of luciferase, chromatin immunoprecipitation, and electrophoretic mobility shift assays showed that TCF-1 could regulate *runx2* directly and *sox9* indirectly

**Fig. 3:**
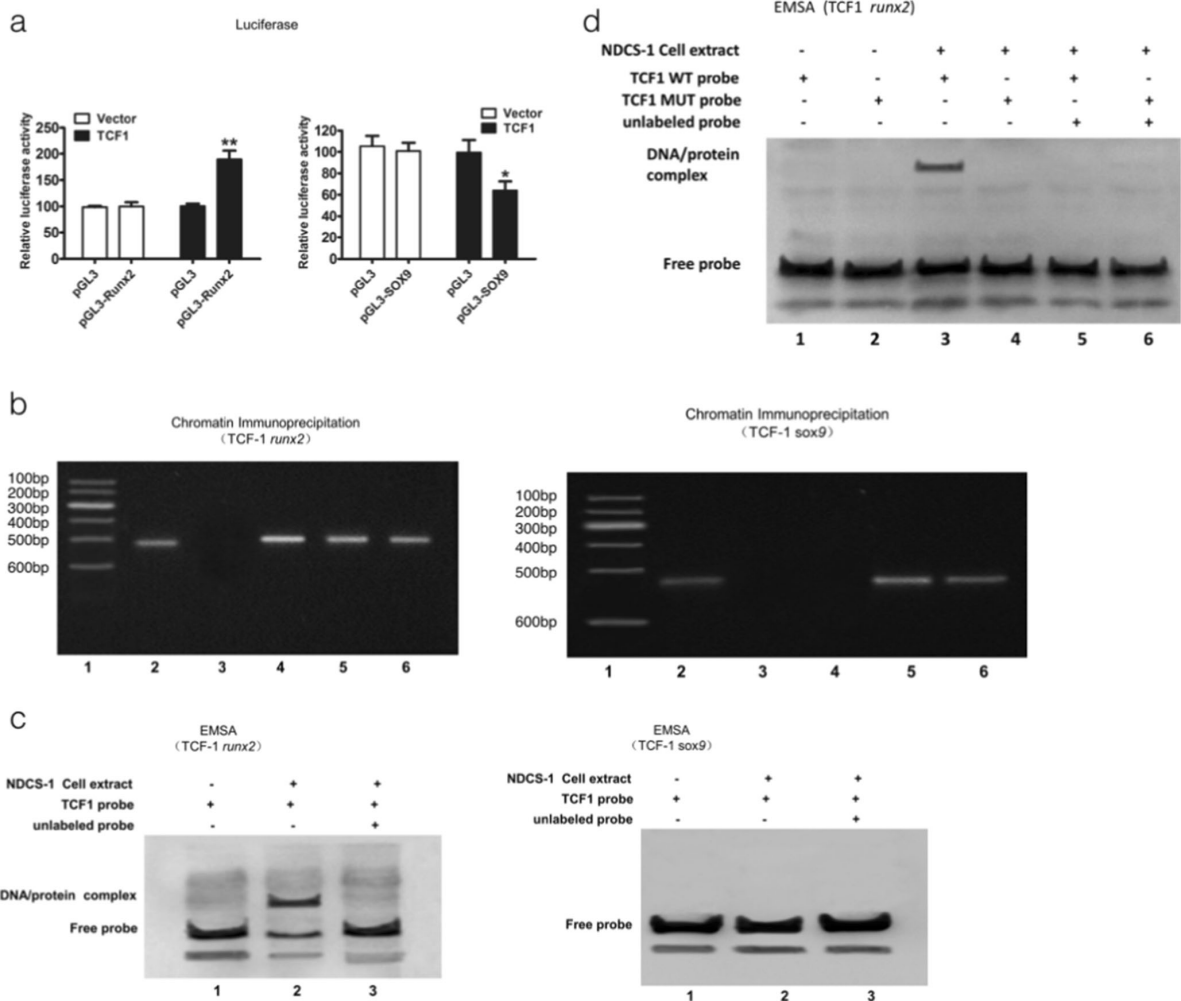
Luciferase, ChIP, and EMSA results showing the specific mechanism that TCF-1 regulates the expression level of *runx2* and *sox9*. Luciferase assay of the *runx2* promoter and *sox9* promoter activity 48 h after co-transfection of Renilla luciferase and the reporter plasmid pGL/RUNX2 or pGL/SOX9 in Hela cell lines transduced with TCF-1 expression plasmid (**a**). The ChIP assay: protein–DNA complexes from NDCS-1 cells were incubated with TCF-1 antibody. The binding of *runx2* DNA was detected by PCR amplification using *runx2*-specific primers. Immunoglobulin G (IgG) antibody and glyceraldehyde-3-phosphate dehydrogenase primers were used in this assay as negative controls. *Lanes 1*–*6* are as follows: *1* DNA marker (MD101), *2* input, *3* IgG, *4* anti-TCF-1, *5* anti-RNA polymerase II, and *6* GAPDH promoter-specific DNA (**b**). The super gel shift assay showed the binding of TCF-1 with RUNX2 DNA in nuclear extracts prepared from chondrosarcoma cell lines. For experimental controls, the labeled RUNX2 probes were incubated alone, with nuclear extracts, in combination with nuclear extracts and unlabeled probe, or in combination with nuclear extracts and IgG antibody (**c**, **d**). *Asterisk* denotes *P* < 0.05; *two* a*sterisks* denote *P* < 0.01

To assess whether TCF-1 can regulate *runx2* and *sox9* expression at the transcriptional level, luciferase reporter assays were performed to verify the TCF-1 effect on *runx2* and *sox9* promoters. TCF-1 overexpression in HeLa cell lines significantly upregulated the *runx2* promoter activity and downregulated the *sox9* promoter activity (Fig. 3a).

The chromatin immunoprecipitation (ChIP) assay was performed to confirm whether TCF-1 could bind directly to *runx2* and *sox9* gene promoters using an anti-TCF-1 antibody. The polymerase chain reaction (PCR) analysis of the TCF-1-immunoprecipitates showed negative results for *sox9* gene but positive results for *runx2* gene in NDCS-1 cell lines (Fig. 3b). Thus, it was hypothesized that TCF-1 might regulate *sox9* indirectly but it can bind directly to the *runx2* gene promoter.

To further determine whether the binding site is the *runx2* promoter, electrophoretic mobility shift assay (EMSA) was performed using an oligonucleotide probe. Nuclear extracts were super-shifted by the anti-TCF-1 antibodies, indicating that TCF-1 binds to the *runx2* promoter. On the contrary, in *sox9* gene, the result was negative. To further confirm whether TCF-1 binds to the *runx2* promoter specifically, another EMSA test was performed using both TCF-1 wild-type probe and mutated probe. Positive results were obtained using the TCF-1 wild-type probe than the mutated probe (Fig. 3c, d). These findings indicated that TCF-1 activated *runx2* gene expression by directly binding to the *runx2* promoter, while it could regulate *sox9* gene expression indirectly.

### The inhibition of TCF-1 by shRNA in NDCS-1 cells inhibited cell division, while the overexpression of TCF-1 by Lentivirus in SW1353 cells promoted cell division. The inhibition of TCF-1 expression in NDCS-1 cells also promoted early apoptosis as well as necrosis

**Fig. 4:**
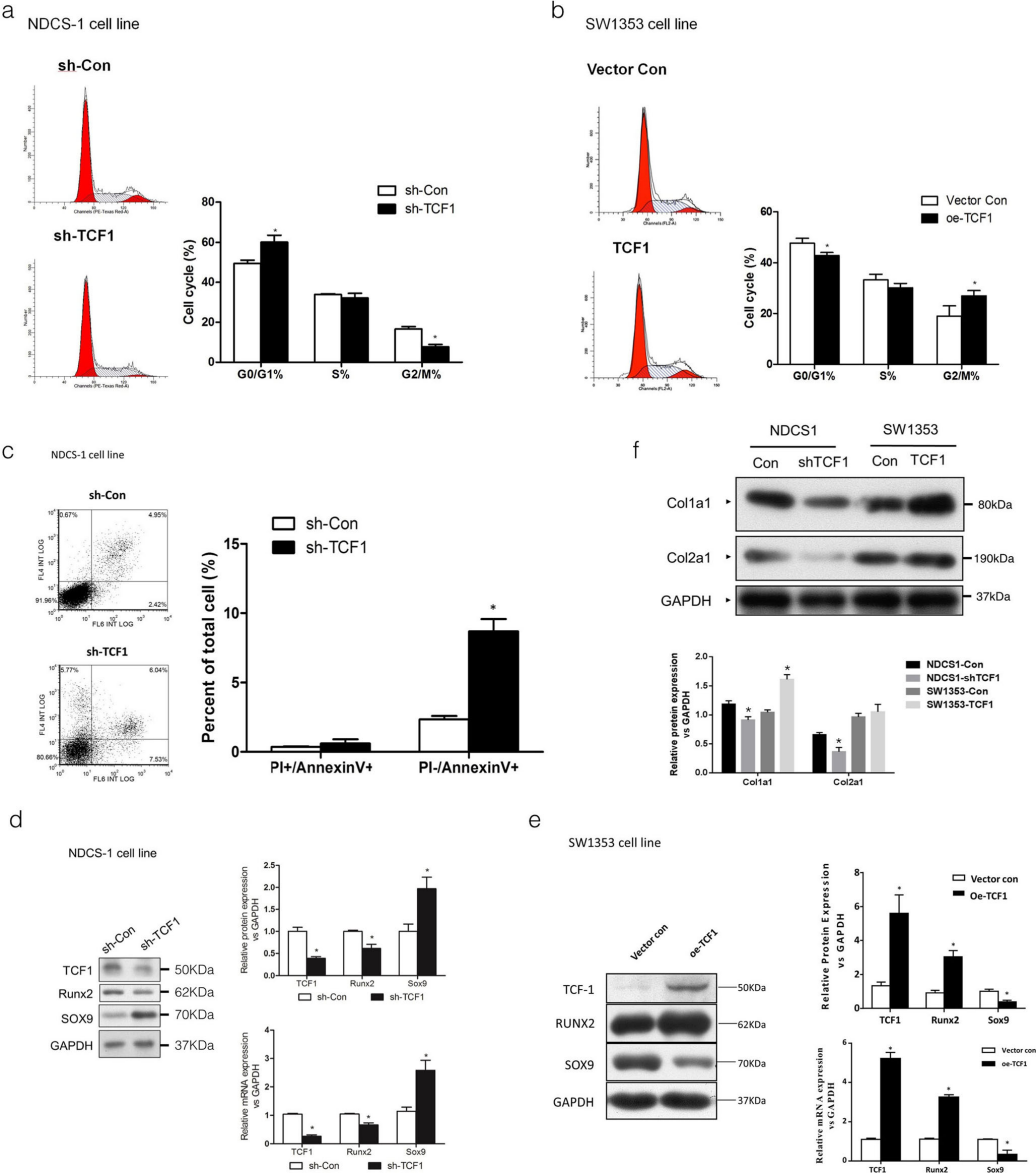
Effects of TCF-1 on the cell cycle, apoptosis, expression level of SOX9 and RUNX2, as well as cell phenotype in NDCS-1 and SW1353 cell lines. Cells were subjected to PI staining and FACS analysis to determine cell cycle profile in down-regulated TCF-1 in NDCS-1 (**a**) or overexpressed TCF-1 in SW1353 cells (**b**). Apoptotic index was determined by the percentage of cells positive for both PI and Annexin V in downregulated TCF-1 in NDCS-1 cells (**c**). Western blot and real-time PCR were performed to evaluate the relative expression of TCF-1, runx2, and sox9 between wild-type and TCF-1 downregulated NDCS-1 cells (**d**) as well as in wild-type and TCF-1 up-regulated SW1353 cells (**e**). Between wild-type NDCS-1, SW1353, TCF-1 downregulated NDCS-1, and TCF-1 overexpressed SW1353 cells, Western blot was performed using col1a1 antibody (Santa Cruz sc-59772) and col2a1 antibody (Santa Cruz sc-52658) to observe the change in the cell phenotype (**f**). Glyceraldehyde-3-phosphate dehydrogenase was used as an internal control for protein equal loading. Data are representatives or expressed as the mean + standard error of mean of three independent experiments. *Asterisk* denotes *P* < 0.05

Here, propidium iodide (PI) staining and flow cytometry were used to determine whether TCF-1 could influence cell division in DDCS as well as CCS cells. In NDCS-1 cell line, the shTCF-1 altered the distribution of dedifferentiated chondrosarcoma cells in different stages of the cell cycle and its progression. The results showed that on treatment with shTCF-1 in NDCS-1 cells, the cells in the G0/G1 phase increased while the cells in the G2/M phase decreased, compared with the control group. In SW1353 cells, the overexpression of TCF-1 could decrease the percentage of cells in the G0/G1 phase and increase the percentage of cells in the G2/M phase, compared with the control group (Fig. 4a, b). In the NDCS-1 cell line, the downregulation of TCF-1 protein promoted early apoptosis as well as necrosis (Fig. 4c). These results showed that the inhibition of TCF-1 by shRNA in NDCS-1 cells inhibited cell division, while the overexpression of TCF-1 by Lentivirus in SW1353 cells promoted cell division.

### The downregulation of TCF-1 in NDCS-1 cell line could alter the expression level of *sox9* and *runx2*, and the phenotype of NDCS-1 cells as well as SW1353 cells changed after a change in the TCF-1 expression level

To confirm whether a change in the TCF-1 expression level could change the expression level of *sox9* and *runx2* subsequently, Western blot analysis and reverse-transcription (RT)-PCR test were performed. The results showed that in NDCS-1 cells, the downregulation of TCF-1 decreased the expression level of *runx2* but increased the expression level of *sox9* (Fig. 4d). While in SW1353 cells, the overexpression of TCF-1 increased the expression level of *runx2* but decreased the expression level of sox9 (Fig. 4e). These results indicated that *sox9* and *runx2* were regulated by TCF-1 in dedifferentiated chondrosarcoma. To further confirm whether the change in phenotype in dedifferentiated chondrosarcoma was caused by TCF-1, Western blot was performed using col1a1 and col2a1 antibodies. It was observed that in DDCS as well as CCS cell lines, a change in the TCF-1 expression level caused a change in the phenotype of cells (Fig. 4f).

### The downregulation of TCF-1 inhibited the growth of dedifferentiated chondrosarcoma in vivo

**Fig. 5:**
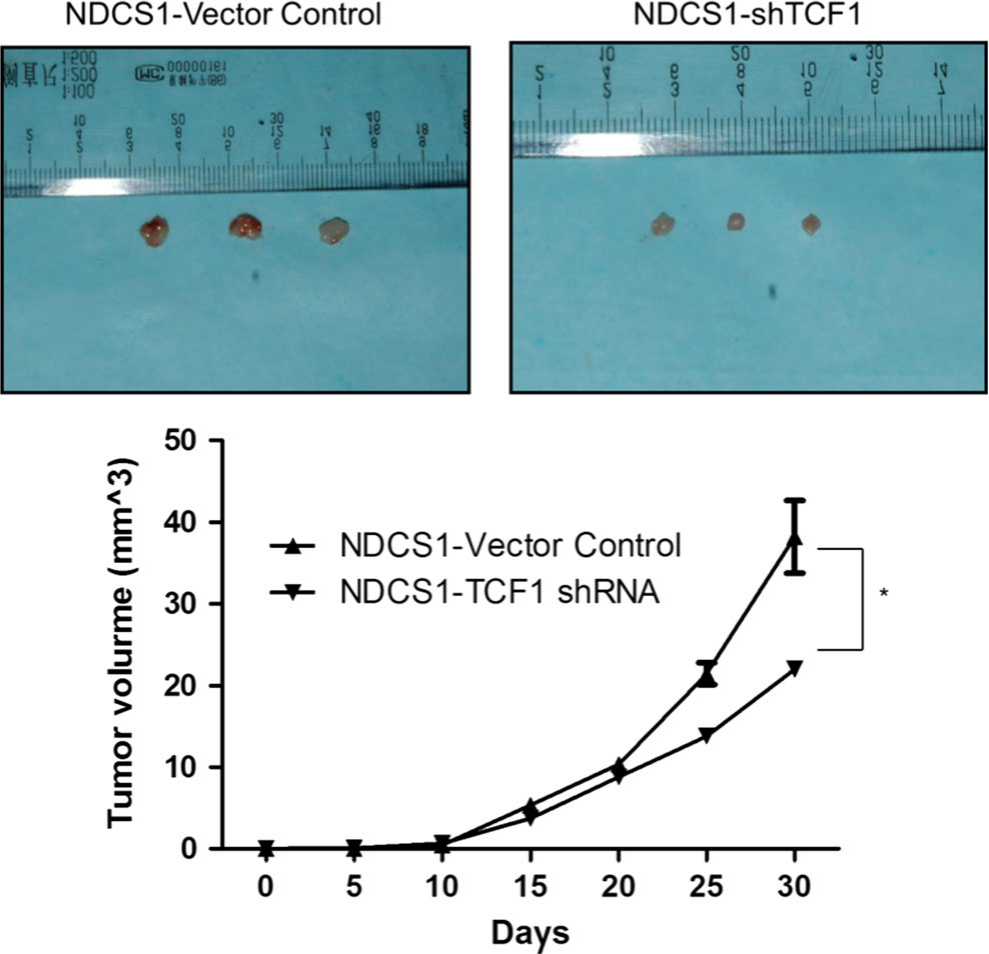
Tumorigenicity ability of wild-type and TCF-1 downregulated NDCS-1 cells. Tumorigenicity was evaluated by nude mice assay. Mice were injected in the right flank subcutaneously with 2 × 10^6^ cells. After 1 month of cell injection, the volume of tumor was calculated (*left panel*) according to the formula: 0.5 × length × width^2^. Data are representatives or expressed as the mean + standard error of mean of three independent experiments. *Asterisk* denotes *P* < 0.05

To confirm the aforementioned in vitro findings, in vivo xenograft models were established. The mouse group injected with NDCS-1, in which TCF-1 was downregulated, had a lower proliferation rate and developed a smaller tumor compared with the negative control group (Fig. 5). The data suggested that the downregulation of TCF-1 inhibits the growth of dedifferentiated chondrosarcoma cells in vivo.

## Methods

### Patients, tissue samples, and follow-up

Eight dedifferentiated chondrosarcoma tissue samples, four grade I CCS tissue samples, and four grade II CCS tissue samples were collected under the protocols approved by the Ethics Committee of Peking University People’s Hospital. Informed consents (written in the light of the ethical guidelines) were obtained from all the patients. A total of 85 formalin-fixed and paraffin-embedded tissue specimens of histopathologically diagnosed chondrosarcoma (including different grades of CCS and DDCS) were obtained from the Department of Pathology and the Musculoskeletal Tumor Center, Peking University People’s Hospital (Beijing, China). The clinical features of these specimens are shown in Table 1. The tissue samples were collected after operation, sectioned (4 μm thickness), and preserved at room temperature until required for the experiment. Clinical and histopathological information was recorded through a retrospective review of the patient records.

### IHC assays for TCF-1

The tissue sections were deparaffinized, and the antigen was retrieved using an antigen retrieval solution. The paraffinized sections were then incubated with a rabbit polyclonal anti-TCF-1 antibody (CST-2203s, dilution, 1:100) overnight at 4 °C, followed by staining with a biotinylated secondary antibody. Known positive controls were included in each experiment, and negative controls were obtained by staining with a nonimmune mouse serum (dilution, 1:100) in phosphate-buffered saline (PBS) instead of the primary antibody. When more than 10 % of tumor cells stained positive for TCF-1, the tumor was considered positive for TCF-1 staining. Immunostaining was evaluated by two independent pathologists who were blinded to the clinical characteristics and outcomes.

### Western blot

Whole-cell and tissue lysates were made using cell lysis buffer (CST) supplemented with proteinase inhibitors. Protein concentrations were determined using the bicinchoninic acid protein assay. Equal amounts of proteins were separated by sodium dodecyl sulfate-polyacrylamide gel electrophoresis and transferred to a polyvinylidene fluoride membrane (Amersham Bioscience). After blocking with 5 % BSA, the primary antibody, human TCF-1 (CST-2203), human Runx2 (CST-12556), human Sox9 (CST-14366), and β-actin (CST-4967) were incubated overnight at 4 °C (dilution 1:1000, 1:1000, 1:1000, and 1:1000, respectively). Then, the appropriate secondary antibody was incubated for 1 h, and the blot was visualized using a Super Signal West Pico Trial Kit (Thermo). The band signals were quantified using ImageJ (Wayne Rasband).

### Cell culture, Lentivirus-mediated shRNA, and transfection

The cell lines used in this research included human DDCS cell line NDCS-1 and human CCS cell line SW1353. The NDCS-1 cell line was kindly provided by Dr Akira Ogose (Niigata University Graduate School of Medical and Dental Sciences, Japan), while the SW1353 cell line was purchased from ATCC. NDCS-1 and SW1353 were grown in 1640 (Gibco) medium and L-15 (Gibco) medium, respectively, supplemented with 10 % fetal bovine serum (Gibco) and 1 % antibiotics. The cultures were maintained at 37 °C in 5 % atmospheric CO_2_.

### Lentivirus particle generation and Lentivirus-mediated shRNA gene overexpression and knockdown

Full-length TCF-1 cDNA and TCF-1 shRNA sequence was subcloned into lentiviral vector pLVTHM. Lentivirus was produced following previously established procedures. Briefly, 293T cells were plated 3 days before transfection. TCF-1 shRNA pLVTHM plasmid was mixed with envelop plasmid (pMD2G) and packaging plasmid (psPAX2) in desired ratio and transfected 293T cells by calcium phosphate. Fresh media were changed 14–16 h post-transfection and supernatant containing Lentivirus were collected 12 h later. Supernatants can be harvested three times every 12 h. Titers of virus were determined using 293T. Three targeting sequences for TCF-1 were used to generate TCF-1 overexpression and shRNA Lentivirus (#1GAGACGCTAGTGGAGGAGTGCAATA; #2 CGGTCCCTTAGTGACAGTGTCTACA; #3 GCGTGTCTACAACTGGTTTGC) and tested for TCF-1 overexpression and knockdown. The #2 sequence had the highest TCF-1 knockdown efficiency and was used to generate stable TCF-1 knockdown NDCS1 cell lines in this study. Efficiency of TCF-1 overexpression and knockdown was confirmed by qPCR and Western blot. The sequence of control shRNA is as follows: 5′-TTCTCCGAACGTGTCAGCTTT-3′.

### Dual-luciferase reporter assay

For the dual-luciferase reporter assay, 1 × 10^5^ cells per well in 24-well plates were seeded the day before transfection. The cells were transfected with 1.0 μg of pGL3-RUNX2-promoter-luciferase or pGL3-SOX9-promoter-luciferase together with 0.2 μg of PRL-TK or 0.4 μg of RhoE-promoter-luciferase. The cells were harvested for 48 h and then detected using the dual-luciferase reporter assay system kit (Promega, WI, USA). The luciferase activity was measured using the Synergy2 instrument (BioTek, VT, USA) equipped with the Gen5 software. The firefly luciferase expression was normalized to Renilla luciferase and reported as relative luciferase activity.

### Chromatin immunoprecipitation assay

The ChIP assay was performed using a EZ ChIP Kit from Millipore. Protein–DNA complexes were immunoprecipitated with TCF-1 antibody, with rabbit serum as a control. Then, the samples were subjected to PCR analysis with the following primers: for RUNX2, promoter 5′-CAGCCACCGAGACCAACA-3′ and 5′-CAGTATGCCTGGAGTACATAGACTT-3′, and for SOX9, promoter 5′-GCTCAAGGTCGATGTGGCG-3′ and 5′-GGTGCGGCTGGTCAGGATT-3′. Sonicated DNA fragment prior to immunoprecipitation was used as an input control, and 5′-TCTCCACACCTATGGTGCAA-3′ and 5′-TTGCCGTGAGTGGAGTCATA-3′ served as the negative control.

### Electrophoretic mobility shift assay

Nuclear extracts from NDCS-1 cells were prepared using a nuclear extraction kit (Thermo Fisher, CA, USA) according to the manufacturer’s protocol. Oligonucleotides were synthesized by Life Technologies (Shanghai, China) and biotinylated using a Biotin 3′-end DNA labeling kit (Thermo Fisher) according to the manufacturer’s protocol. The sequences used were 5′-TTGTTTTGTTTCTTTGCTTTTCACATGT-3′ and its complementary sequence. The mutant sequences were 5′-TTGTTTTGTTTGGGCCCTTTTCACATGT-3′. Binding reactions were performed using a LightShift Chemiluminescent EMSA kit (Thermo Fisher) according to the manufacturer’s protocol. Briefly, nuclear protein samples (10 μg) were incubated with Biotin-labeled TCF-1 oligonucleotide for 20 min at room temperature in reaction buffer [2 mM MgCl_2_, 0.1 mM ZnSO_4_, 7.5 % glycerol, 10 ng/μL of poly(dAdT) (Sigma), and 1 μg of bovine serum albumin] and probe alone as a nonspecific competitor. The analyzed DNA–protein complexes were electrophoresed in 4 % polyacrylamide gels containing 0.5× TBE buffer, and then transferred to a positively charged nylon membrane (Bio-Rad, CA, USA) using a semi-dry transfer blotter. The image was visualized and scanned using a SuperSignal West Pico chemiluminescent substrate kit (Pierce, IL, USA).

### In vitro cell invasiveness tests

#### Cell proliferation assay

The cell proliferation assay was performed using a Cell Counting Kit-8 (Dojindo, Japan), a redox assay similar to the 3-(4,5-dimethylthiazol-2-yl)-2,5-diphenyltetrazolium bromide assay according to the manufacturer’s protocol.

#### Apoptosis analysis

After washing the cells three times with ice-cold PBS, they were resuspended in binding buffer, stained with Annexin V-FITC (BD Biosciences) and PI (Sigma-Aldrich), and analyzed by flow cytometry (BD Biosciences).

#### Cell cycle assay

The cells were seeded 16 h before analysis (20 % of confluence), collected, and stained with 200 μg/mL of PI, 0.1 % sodium azide, 0.1 % Triton X-100, and 10 μg/mL of RNAses for 2–4 h. Single cells were analyzed for subG1, S, and G2 peaks using fluorescence-activated cell sorting array (BD Bioscience). The analysis was performed using the FlowJo software.

#### Invasion assay

The migration of the cells was measured by counting the number of cells that migrated through Transwell inserts with 3-μm pores, as described previously. The cells were trypsinized, and 200 μL of cell suspension (1 × 10^6^ cells/mL) from each treatment was added in triplicate wells. After 24-h incubation, the cells that had migrated through the filter into the lower wells were quantitated using the gentian violet assay and expressed as the total number of cells in the lower wells.

#### Colony formation assay

A total of 500 cancer cells per well were seeded in a six-well plate. After culture for 2–3 weeks, the cell culture was terminated and the plate was washed with PBS twice. The cells were fixed with 4 % paraformaldehyde for 15 min. Then, the cells were incubated with Trypan blue for 10 min, and the staining solution was washed.

#### Western blot

Standard Western blot was performed to measure the expression level of TCF-1, Runx2, and Sox9 proteins as described previously.

#### RT-qPCR assay

Total RNA was extracted using Trizol reagent (Invitrogen, CA, USA), and the reverse-transcription reactions were performed using an M-MLV Reverse Transcriptase kit (Invitrogen). Real-time PCR was performed using a standard SYBR Green PCR kit (Toyobo, Osaka, Japan). The primers used in RT-PCR were as follows: TCF-1 forward, 5′-CCCACCAAGCAGGTCTTCAC-3′; TCF-1 reverse, 5′-AAGGTCTCGATGACGCTGTG-3′; RUNX2 forward, 5′-ACAGTGACACCATGTCAGCA-3′; RUNX2 reverse, 5′-TCGGCGATGATCTCCACCAT-3′; SOX9 forward, 5′-GGACCACCCGGATTACAAGT-3′; and SOX9 reverse, 5′-AAGATGGCGTTGGGGGAGAT-3′. mRNA levels were calculated as the fold change of control.

#### Xenograft tumorigenicity assays

Nude BALB/c mice (6 weeks old, female, 20 to 30 g body weight) were purchased from Shanghai SLAC Laboratory animal Co. Ltd. (Shanghai, China) and maintained under pathogen-free conditions. All animal experiments proceeded according to the standards of Institutional Animal Care and Use Committee and performed according to an established protocol approved by the Ethics Committee of Peking University People’s Hospital. The cells (2 × 10^6^) were introduced by subcutaneous implantation in PBS into 6-week-old immunodeficient nude mice. The mice were sacrificed 30 days after tumor implantation. The volumes of tumors were measured every 5 days and calculated as *V* = *a* × *b* ^2^ × π/6.

#### Statistical analysis

All statistical analyses were performed using the SPSS19.0 software package (SPSS Inc., Chicago, IL, USA). The relationship between patient survival and indicated protein levels was assessed using the Kaplan–Meier analysis. The correlation between protein levels and clinicopathological tumor grading was analyzed using standard *χ* ^2^ test. Student’s *t* test was used to specify the differences with a *P* < 0.05.

## Discussion

Dedifferentiated chondrosarcoma is a special variant of chondrosarcoma, most commonly seen in cases with local recurrence. It is a kind of highly malignant tumor with a high local recurrence rate and distal metastasis rate. Till now, no effective therapy is available for this tumor except surgery [7]. With regard to the origin of the two different cell components in DDCS, Bridge et al. showed that they arise from the same precursor. However, the reason for the difference in differentiation is still unknown [24]. Most previous researches about DDCS were case reports rather than researches on mechanism. This study focused on the pathway and molecules that participate in DDCS occurrence, which is vital to explore a novel therapy for this tumor.

*sox9* and *runx2* are two key factors that participate in bone and cartilage formation [11]. *sox9* participates in the maturation of chondrocytes. It can regulate the expression of *col2a1* gene, which is a specific marker of chondrocytes [25–30]. It also participates in the occurrence of chondrosarcoma [19, 20]. *runx2* gene is a key point that regulates the differentiation of osteocytes from mesenchymal cells [31, 32]. This gene is regulated by a number of pathways such as Wnt signaling and bone morphogenetic protein (BMP) signaling [11]. Also, *runx2* participates in the occurrence of many tumors [33–38]. In a previous study, Xiaodong et al. proved a higher expression of *runx2* gene and lower expression of *sox9* gene in DDCS cell lines compared with CCS cell lines. However, what results in the different expression of *sox9* and *runx2* remains unknown.

Canonical Wnt signaling plays a significant role in regulating a number of biological processes [10, 13]. Many researches show that aberrant Wnt signaling participates in many kinds of diseases such as digestive tract tumors or osteoporosis [14–18]. TCF-1 is a member of TCF/LEF family, which is the downstream effector of canonical Wnt signaling. Although the role of TCF/LEF family has been studied in many tumors such as gastrointestinal tumor or leukemia, the role of TCF-1 in dedifferentiated chondrosarcoma was not explored yet. Day et al. reported that Wnt signaling determines chondrogenesis or ossification via *sox9* and *runx2* [11]. Gaur et al. reported that TCF-1 participates in osteogenesis via *runx2* gene as well as Smad1/5 [12]. Miclea et al. found that Wnt/TCF can regulate the expression of *sox9* [39]. This study proposed a novel mechanism that TCF-1 can regulate *runx2* and *sox9* in DDCS, and it is closely related to the patients’ prognosis. Using ChIP and EMSA technologies, it was found that TCF-1 can upregulate *runx2* by binding to its promoter region directly, which is consistent with other previous studies. However, the ChIP and EMSA findings showed that TCF-1 could downregulate *sox9*, but it could not bind to its promoter region, indicating the existence of some other molecules that mediate the TCF-1 effect. This needs further investigation to figure out the specific mechanism.

Other important pathways that control the bone or cartilage formation are the BMP/Smad pathway [40, 41] and noncanonical Wnt pathway. The BMP/Smad pathway also participates in the formation of many tumors [42]. It is reported that the BMPR2 expression level in DDCS is much higher than that in CCS, which may have something to do with the DDCS formation. Jiao et al. showed a higher pathologic grade of chondrosarcoma and a higher expression level of BMPR2. Also, its expression level is related to the patients’ prognosis [43]. The noncanonical Wnt pathway consists of many subfamilies, one of which is the PLC-Ca^2+^-TAK1-NLK pathway. Many studies reported that this pathway could repress the growth of many tumors [44–47]. It is also reported that nemo-like kinase (NLK), a member of the PLC-Ca^2+^-TAK1-NLK cascade, can regulate the canonical Wnt pathway and BMP/Smad pathway through phosphorylation of TCF-1 protein as well as Smad protein [10, 48, 49]. It is considered that DDCS formation may be a cross-talk effect of BMP/Smad pathway, canonical Wnt pathway, and noncanonical Wnt pathway. This needs to be investigated further.

In conclusion, this study proved that TCF-1 participates in the dedifferentiation of DDCS, which may be mediated by *runx2* gene and *sox9* gene. The results suggest that TCF-1 can be of important prognostic value. More importantly, it can be a promising therapeutic target for DDCS patients.
